# Viral and bacterial co-infection in hospitalised children with refractory *Mycoplasma pneumoniae* pneumonia

**DOI:** 10.1017/S0950268818000778

**Published:** 2018-07-04

**Authors:** Xinxing Zhang, Zhengrong Chen, Wenjing Gu, Wei Ji, Yuqing Wang, Chuangli Hao, Yanyu He, Li Huang, Meijuan Wang, Xuejun Shao, Yongdong Yan

**Affiliations:** 1 Department of Respiration, Children's Hospital of Soochow University, Suzhou 215003, China; 2 Department of Clinical Laboratory, Children's Hospital of Soochow University, Suzhou 215003, China

**Keywords:** Children, co-infection, RMPP

## Abstract

To investigate the impact of viral and bacterial co-infection in hospitalised children with *Mycoplasma pneumoniae* pneumonia (RMPP). Retrospective analysis of 396 children with RMPP in our hospital admitted between 1 January 2011 and 31 December 2016 was performed. Nasal aspirate samples were collected for pathogen detection and clinical data were collected. We analysed clinical characteristics, lung imaging characteristics and pathogenic species among these children. Of the 396 RMPP cases, 107 (27.02%) had co-infection with other pathogen, with *Streptococcus pneumoniae*, *Haemophilus influenzae* and *Staphylococcus aureus* being the most common bacteria of infection and human bocavirus (HBoV), human rhinovirus, respiratory syncytial virus being the most common viruses of infection. Children with co-infection were younger than that with single infection (*P* = 0.010). Children with both virus and bacteria co-infection had been the youngest (*P* = 0.040). Children with co-infection had a longer fever process, higher leukocyte count, higher C-reactive protein compared with single infection (*P* < 0.05). Children with co-infection had a higher percentage of pnemothorax and diffuse large area of inflammation in chest X-ray manifestation compared with children with single infection (*P* < 0.05). *S. pneumonia* and HBoV was the leading cause of co-infection in RMPP. Co-infections led to more disease severity in children with RMPP compared with single infections.

## Introduction

Pneumonia is the leading cause of childhood mortality, with nearly 1.3 million deaths each year. *Mycoplasma pneumoniae* (MP) is a leading cause of community-acquired pneumonia (CAP) in children and young adults [1, 2]. Studies showed that *M. pneumoniae* was detected in 30% of paediatric CAP and in over 50% among children aged 5 years or older [3].

MP infection is usually a self-limited disease. However, previous studies have shown that MP infection can develop into a severe life-threatening disease in rare cases, such as refractory *Mycoplasma pneumoniae* pneumonia (RMPP), acute respiratory distress syndrome, necrotising pneumonitis and fulminant pneumonia [4, 5]. Nowadays, the underlying mechanisms of RMPP are still uncertain. Studies showed that the macrolide-resistant *M. pneumoniae* infection and excessive immunological inflammation may play important roles in the occurrence and development of RMPP [6, 7]. However, there is a scarcity of studies investigating co-infections of *Mycoplasma pneumoniae* pneumonia (MPP) especially RMPP in children [8].

The purpose of this study was to investigate the impact of viral and bacterial co-infection in hospitalised children with RMPP. In this study, we encountered several cases of RMPP whose lung inflammation were difficult to absorb which required investigation using fibre optic bronchoscopy. We retrospectively analysed all children with RMPP over a 6-year period to study the clinical features, laboratory data and co-infections of these children.

## Methodology

### Study patients

All participants’ parents or guardians were given written informed consent before study enrolment. Potential ethical problems related to this study were examined and approved by ethics committee review of Soochow University. We retrospectively collected the data of patients with RMPP who were admitted to the Department of Respiratory Medicine in the Children's Hospital of Soochow University between 1 January 2011 and 31 December 2016. The exclusion criteria for our study were (1) patients with congenital heart diseases, heredity metabolic diseases, neurological disorders, bronchopulmonary dysplasia and immunodeficiency; (2) those with incomplete clinical data; and (3) those in the convalescent stage of the disease.

### Definitions

MPP was confirmed when (1) a pulmonary infiltrate on a chest radiograph was present in combination with fever, cough or auscultatory findings that were consistent with pneumonia and (2) the presence of IgM antibodies together with *M. pneumoniae* DNA. RMPP were defined as cases showing clinical and radiological deterioration despite appropriate antibiotic therapy for 7 days or more [9].

### Data collection

Demographic, clinical information, laboratory data, radiological were retrospectively collected from the records of all children. Nasal aspirate samples were collected for microbiological analysis.

### Nasal aspirate samples collection

Nasal aspirate samples were obtained from each patient within 24 h after admission, using a sterile plastic catheter briefly inserted into the lower pharynx via the nasal cavity, for detection of common viruses.

### MP serology

The specific IgM antibodies against *M. pneumoniae* were detected in 2 ml of acute phase (on admission) and convalescent-phase (on discharge) patient serum using a commercial ELISA kit (SERION ELISA classic *M. pneumoniae* IgM; Institute Virion/Serion, Würzburg, Germany), in accordance with the manufacturer's instructions and as described previously. The test cut-off value was 0.5 × mean optical density (OD) of the kit control serum, as indicated in the insert. A positive IgM antibody reaction was defined as > 1.1 S/CO.

### Fluorescent quantitation (FQ) polymerase chain reaction (PCR) for *M. pneumoniae* gene detection

A 16S rRNA gene PCR procedure was used for the detection of *M. pneumoniae*. In brief, one of the equally divided samples of nasal aspirate was shaken for 30 s and centrifuged at 15 000 × g for 5 min. The sediment was collected and DNA extracted from a 400-μl sample in accordance with the manufacturer's instructions. The DNA was then amplified using PCR primers and probes. Primers and probes were synthesised using the following sequences: *M. pneumoniae* -F: 50-GCAAGGGTTCGTTATTTG-30; *M. pneumoniae* -R: 50-CGCCTGCGCTTGCTTTAC-30 (344 bp); *M. pneumoniae* -probe: 50-AGGTAATGGCTAGAGTTTGACTG-30 (141 bp). FQ-PCR was performed using an iQ5TM BIO-iCycler (Bio-Rad, California, USA), and the cycling conditions were as follows: 2 min at 37 °C; 10 min at 94 °C, and 40 cycles of 10 s at 94 °C, 30 s at 55 °C, and 40 s at 72 °C. Quantification curves were plotted using several concentrations of standard control samples, which were purchased from Daan Gene Co. Ltd (Guangzhou, China). For each assay, a negative quality control, a critical quality control, a positive quality control, and four positive quantity controls (10^5^, 10^6^, 10^7^ and 10^8^ copies/ml) were used. The results of were considered positive if an exponential fluorescence curve could cross the assigned threshold at Ct < 38.0.

### Bacteria culture

Bacteria were tested by inoculating nasal aspirate samples on blood plates that were read after incubating for 18–20 h. If bacterial growth >10^4^ colony forming units/ml, it was considered significant. Morphology selection was depended on experienced clinical laboratory physician, and colonies were identified by specific tests where necessary such as optochin sensitivity and bile solubility tests for *Streptococcus pneumoniae*, tests for the requirement of growth factors X, V and XV for *Haemophilus influenzae*.

### Seven common respiratory viruses detection

Ten types of viruses and bacteria were tested in nasal aspirate samples from the RMPP children. Viruses including respiratory syncytial virus (RSV), adenovirus (ADV), influenza virus (A, B) (IV-A and IV-B) and parainfluenza virus (1, 2, 3) (Pinf-1, Pinf-2, Pinf-3) were investigated by immunofluorescence tests using D3 Ultra™ Respiratory Virus Screening and LD Kit (Diagnostic Hybrids, Ohio, USA). A positive result was defined as over five inclusion bodies analysed under a fluorescence microscope.

### Detection the human rhinovirus (HRV) gene by RT–PCR

The primer sequences for HRV were HRV-F: TGG ACA GGG TGT GAA GAG C；HRV-R: CAA AGT AGT CGG TCC CAT CC；HRV PROBE：FAM-TCC TCC GGC CCC TGA ATG-TAMRA. Nasal aspirate samples RNA was extracted as described above, and HRV-RNA was detected by real-time fluorescent PCR. The cyclic temperature settings were 95 °C 5 min，95 °C 15s 60 °C 30s amplified by 40 cycles.

### Detection of the human metapneumovirus (hMPV) gene by RT-PCR

The primer sequences for hMPV were 5′-AACCGTGTACTAAGTGATGCACTC-3′; antisense, 5′-CATTGTTTGACCGGCCCCATAA-3′RNA was extracted from nasal aspirate samples specimens using Trizol (Invitrogen, USA). cDNA was synthesised by reverse transcription. The cyclic temperature settings were 94 °C, 30 s; 55 °C, 30 s; 68 °C, 30 s; amplified by 45 cycles with the last at 68 °C for 7 min. hMPV was assayed by fluorescent real-time PCR (BIO-RAD iCycler). The cyclic temperature settings were 94 °C, 30 s; 56 °C, 30 s; 72 °C, 30 s; amplified, 40 cycles.

### Detection the human bocavirus (hBoV) gene

The primer sequences for hBoV were 5′-TGACATTCAACTACCAACAACCTG-3′; hBoV-R:5′-CAGATCCTTTTCCTCCTCCAATAC-3′; hBoV-probe: AGCACCACAAAACACCTCAGGGG-TAMRA; Nasal aspirate samples DNA was extracted as described above, and hBoV-DNA was detected by real-time fluorescent PCR. The cyclic temperature settings were 94 °C, 30 s; 56 °C, 30 s; 72 °C, 30 s; amplified by 40 cycles.

### Statistical analysis

Measurement data were reported as mean ± standard deviation. The differences between 2 groups were compared using the nonparametric Mann–Whitney two-sample *U* test for unpaired data. The differences among three or more groups were compared using the nonparametric Mann–Whitney *U* test. Enumeration data are shown as rate, determined by chi-squared test and Fisher's exact test. SPSS version 18.0 software was used in the data analysis. A *P*-value <0.05 was considered significant.

## Results

### Population

A total of 396 RMPP cases were analysed in the present study. Among them 289 (72.98%) cases were single infected with *M. pneumoniae* and 107 (27.02%) cases were positive for at least one bacterial or virus pathogen in addition to MP. The mean age of the patients was 5.53 ± 2.80 years. Among these 396 cases, 247 cases had positive *M. pneumoniae* DNA (188 cases in single infection group and 59 cases in co-infection group) and 200 cases had the presence of IgM (158 cases in single infection group and 42 cases in co-infection group).

### Clinical symptoms and laboratory examination compared with RMPP children with single infection and co-infection

Children with co-infection were younger than that with single infection (*P* = 0.010). Significant differences were observed in Fever process, leukocyte count, C-reactive protein>38 mg/l and C-reactive protein>100 mg/l between single and co-infections (*P* < 0.05). Although the co-infection group had a longer course of hospital stay, higher percentage of neutrophils, higher number of platelet and higher rate of elevated serum alanine aminotransferase (ALT), there was no significant difference between the two group (0.05 < *P* < 0.10). There was no significant difference in gender, lymphocyte, serum lactate dehydrogenase and creatine kinase muscle B (CKMB) between patients with single infections and those who with co-infection (*P* > 0.10) (Table 1).

**Table 1.** Clinical characteristics of RMPP children hospitalized with *M. pneumoniae* single infection or co-infection with other pathogens

Characteristic	Single infection	Co-infection	*P* value
Number	289	107	
Males *n* (%)	153 (52.9)	57 (53.3)	1.000
Ages (year)	5.75 ± 2.84	4.94 ± 2.59	0.010
Hospital stay (day)	10.56 ± 4.34	11.72 ± 5.21	0.081
Fever process (day)	12.22 ± 4.18	13.40 ± 3.92	0.000
Laboratory findings			
Leukocyte count (× 10^9^/l)	9.19 ± 4.40	11.41 ± 5.20	0.000
Neutrophil (%)	63.46 ± 15.28	66.13 ± 16.07	0.084
Lymphocyte (%)	27.67 ± 13.94	28.16 ± 15.76	0.946
Platelet (×10^9^/l)	321.00 ± 107.21	340.84 ± 112.19	0.057
C-reactive protein>8 mg/l *n* (%)	202 (69.9)	79 (73.8)	0. 533
C-reactive protein>38 mg/l *n* (%)	71 (24.6)	38 (35.5)	0.042
C-reactive protein>100 mg/l *n* (%)	19 (6.6)	14 (13.1)	0.043
Elevated serum LDH *n* (%)	201 (69.6)	84 (78.5)	0.101
Elevated serum CKMB *n* (%)	24 (8.3)	12 (11.2)	0.431
Elevated serum ALT *n* (%)	44 (15.2)	9 (8.4)	0.096

### Lung imaging characteristics compared with RMPP children with single infection and co-infection

Children with co-infection had a higher percentage of pnemothorax and diffuse large area of inflammation in chest X-ray manifestation and had significant difference compared with children with single infection (*P* < 0.05). There was no significant difference in lobar pneumonia, pulmonary atelectasis and pleural effusion between two groups (Table 2).

**Table 2.** Lung imaging characteristics of RMPP children hospitalised with *M. pneumoniae* single infection or co-infection with other pathogens [*N* (%)]

Lung imaging characteristics	Single infection *N* = 289	Co-infection *N* = 107	*P* value
Lobar pneumonia	251 (86.9)	96 (89.7)	0.496
Pulmonary atelectasis	78 (27.0)	30 (28.0)	0.899
Pleural effusion	10 (3.5)	7 (6.5)	0.261
Pneumothorax	1 (0.3)	5 (4.7)	0.006
Diffuse large area of inflammation	1 (0.3)	8 (7.5)	0.000

### Virus and bacteria diagnosis

Among those children who infected with other pathogens in addition to *M. pneumoniae*, 61 (57.0%) children had only detected bacteria positive, with *S. pneumonia*, *H. influenzae* and *Staphylococcus aureus* being the most common source of infection. Thirty-five (32.7%) children had only detected virus positive, hBoV, HRV and RSV being the most common source of infection. Other 11 children (10.3%) had both virus and bacterial co-infection with *M. pneumoniae*. The data were presented in Table 3.

**Table 3.** Other pathogens detected from 107 patients with RMPP

Co-infection with bacteria	cases	Co-infection with virus	Cases
*S. pneumoniae*	43	HBoV	9
*H. influenzae*	9	HRV	8
*S. aureus*	4	RSV	6
*K. pneumoniae*	1	Pinf-3	5
*E. coli*	1	ADV	3
*P. aeruginosa*	1	IV-A	2
*Baumanii*	1	Pinf-3 + HBoV	1
*S. pyogenes*	1	IV-A + HBoV	1

Co-infection with both bacteria and virus		cases	
*S. pneumoniae* + RSV		3	
*S. pneumoniae* + IV-A		3	
*S. pneumoniae* + ADV		1	
*S. pneumoniae* + HRV		1	
*H. influenzae* + Pinf-3		1	
*S. pneumoniae* + *S. aureus* + IV-A		1	
*S. pneumoniae* + ADV + HBoV		1	

### Clinical symptoms and laboratory examination compared among RMPP children with co-infection

We grouped the children according to the pathogens they detected and analysed the clinical symptoms and laboratory examination among them. Children co-infection with both bacteria and virus were the youngest and were significantly younger than children co-infection with bacteria (*P* = 0.040). There was no significantly different in other clinical characteristics among the three groups (*P* > 0.05) (Table 4).

**Table 4.** Clinical characteristics of RMPP children hospitalised with co-infection with other pathogens

Characteristic	Co-infection with bacteria	Co-infection with virus	Co-infection with both bacteria and virus	*P* value
Number	61	35	11	
Males *n* (%)	33 (54.1)	19 (54.3)	5 (45.5)	0.860
Ages (year)	5.21 ± 2.61	4.93 ± 2.67	3.48 ± 1.90*	0.127
Hospital stay (d)	11.98 ± 5.55	10.80 ± 3.55	13.18 ± 7.39	0.740
Fever process (d)	13.34 ± 4.25	13.34 ± 3.51	13.91 ± 3.45	0.607
Laboratory findings				
Leukocyte count (×10^9^/l)	11.80 ± 5.66	11.14 ± 4.94	10.12 ± 2.88	0.746
Neutrophil (%)	67.93 ± 15.47	62.68 ± 16.50	67.15 ± 17.67	0.276
Lymphocyte (%)	25.47 ± 14.63	32.13 ± 16.27*	30.45 ± 18.59	0.128
Platelet (×10^9^/l)	327.41 ± 123.52	356.34 ± 95.78	366.00 ± 89.58	0.233
C-reactive protein>8 mg/l *n* (%)	47 (77.0)	26 (74.3)	6 (54.5)	0.294
C-reactive protein>38 mg/l *n* (%)	26 (42.6)	9 (25.7)	3 (27.3)	0.208
C-reactive protein >100 mg/l *n* (%)	12 (19.7)	2 (5.7)	0 (0.0)	0.059
Elevated serum LDH *n* (%)	46 (75.4)	29 (82.9)	9 (81.8)	0.667
Elevated serum CKMB *n* (%)	5 (8.2)	5 (14.3)	2 (18.2)	0.490
Elevated serum ALT *n* (%)	5 (8.2)	3 (8.6)	1 (9.1)	0.994

*P < 0.05 compared with co-infection with bacteria group.

## Discussion

This retrospective study investigated the impact of the mixed viral and bacterial co-infection on the presentation and outcome of children of RMPP. A total of 396 children with RMPP were infected with another pathogen. *S. pneumoniae* was the leading cause of bacterial co-infection and hBoV was the leading cause of virus co-infection. Co-infections led to more disease severity in children with RMPP compared with single infections.

Studies have shown that mixed viral-bacterial aetiology is common in children with lower respiratory tract infections [10]. About one-quarter of the paediatric RMPP cases in this study were associated with positive viral and/or bacterial detection. Respiratory microorganisms were detected in all cases. Among these RMPP children who co-infected with other pathogens, more than half of them were co-infected with bacteria, and one-third were co-infected with the virus. These data are consistent with previous aetiological studies of paediatric MPP [11] but higher than other reports from China [8, 12]. This difference might be related to the local environment, climate and pathogenic epidemiology. *S. pneumoniae* was the major bacteria identified in co-infected children, followed by *H. influenzae* and *S.aureus*. HBoV, HRV and RSV were the most common source of virus co-infection. These data are consistent with previous aetiological studies of co-infection in MPP [8, 11, 12].

The present study showed that children with co-infection were younger than that with single infection, and children co-infected with both virus and bacteria were the youngest. This suggested that young children were prone to mixed infection. RMPP children who had co-infection with other pathogen had a longer process of fever, higher leukocyte count, higher C-reactive protein and a higher incidence of pneumothorax or diffuse large area of inflammation in the lung. When studying severe CAP in adults, Guillaume *et al.* found that viral-bacterial co-infection is associated with an impaired presentation and a complicated course [13]. We identified the virus and/or bacteria co-infected RMPP patients to be at risk of severe course, so further studies might explore potential benefits of the early use of antibiotics and antiviral drugs in severe RMPP cases.

There were several limitations to our study. First, this is a monocentre study, so the generalisation of our results should be cautious. Second, as this was a retrospective study, select bias might exists and further prospective studies are potentially needed.

## Conclusion

Virus and bacterial co-infections are relatively common in RMPP. *S. pneumoniae* and hBoV are the most common cause of co-infection in RMPP. Co-infections led to more disease severity in children with RMPP compared with single infections.
